# Tumor-derived exosomes, myeloid-derived suppressor cells, and tumor microenvironment

**DOI:** 10.1186/s13045-019-0772-z

**Published:** 2019-08-22

**Authors:** Xinyu Tian, Han Shen, Zhiyang Li, Tingting Wang, Shengjun Wang

**Affiliations:** 1 Department of Laboratory Medicine, Nanjing Drum Tower Hospital, Nanjing University Medical School, Nanjing, China; 2 Department of Laboratory Medicine, Affiliated Wuxi People’s Hospital of Nanjing Medical University, Wuxi Children’s Hospital, Wuxi, China; 3 Department of Laboratory Medicine, The Affiliated People’s Hospital, Jiangsu University, Zhenjiang, China; 4 Department of Immunology, Jiangsu Key Laboratory of Laboratory Medicine, School of Medicine, Jiangsu University, Zhenjiang, China

**Keywords:** Tumor-derived exosomes, Myeloid-derived suppressor cells, Tumor microenvironment, Immunosuppression, Intercellular communication

## Abstract

Plenty of immune cells infiltrate into the tumor microenvironment (TME) during tumor progression, in which myeloid-derived suppressor cells (MDSCs) represent a heterogeneous population of immature myeloid cells with immunosuppressive activity. Tumor cells and stromal cells facilitate the activation and expansion of MDSCs in TME via intercellular communication, and expanded MDSCs suppress anti-tumor immune responses through direct and indirect mechanisms. Currently, exosomes, which are a kind of extracellular vesicles (EVs) that can convey functional components, are demonstrated to participate in the local and distal intercellular communication between cells. Numerous studies have supposed that tumor-derived exosomes (TEXs), whose assembly and release can be modulated by TME, are capable of modulating the cell biology of MDSCs, including facilitating their activation, promoting the expansion, and enhancing the immunosuppressive function. Therefore, in this review, we mainly focus on the role of TEXs in the cell-cell communication between tumor cells and MDSCs, and discuss their clinical applications.

## Background

Exosomes are EVs with a double membrane structure that can be released by almost all cells and transport functional components into recipient cells [1]. Relying on the transmission of lipids, proteins, and nucleic acids, exosomes change the phenotype and function of recipient cells. Hence, exosomes have now been implicated in numerous biological and pathological processes, including cancer [2–4]. In cancer progression, exosomes released by tumor cells and stromal cells contribute to the initiation and migration of cancer. Additionally, TEXs have been revealed to enhance the development and suppressive function of MDSCs in recent studies [5, 6].

During tumorigenesis, the co-evolution of malignant cells and their direct environment results in the initiation of a tumor. Structures, including vascular vessels, immune infiltrates especially MDSCs, fibroblasts, and extracellular matrix (ECM), constitute the TME which is necessary for cancer progression [7]. MDSCs are identified as immature myeloid cells with immunosuppressive activity in TME [8, 9]. In tumor progression, molecules from TME accelerate the activation, expansion, and immunosuppression of MDSCs. Meanwhile, the expanded and activated MDSCs enhance the proliferation, angiogenesis, migration, and immune escape of cancer. MDSCs infiltrating into TME account for the resistance towards cancer immunotherapy and are responsible for the poor prognosis of chemotherapy [10]. Nowadays, the nature of MDSCs has been revealed gradually, and MDSCs are emerging as a crucial regulator of anti-tumor immune responses [11–14]. Moreover, abundant clinical studies have supposed that MDSCs can act as a valuable predictive marker reflecting the cancer progression, and extensive efforts in developing therapies targeting MDSCs are ongoing [15, 16]. All these imply the critical role of MDSCs in TME during cancer progression.

As mentioned above, exosomes from cancer cells, whose formation and release can be modulated by TME, are emerging as a new modulator of the cell biology of MDSCs [17]. In this review, we highlight the most recent advances on the role of TEXs in modulating the cell biology of MDSCs in TME, with an emphasis on accurate regulatory mechanisms and clinical applications.

## TEXs

Exosomes are a kind of EVs that can be secreted from all cells. Exosomes are identified based on the size (50–100 nm in diameter), density (1.13–1.19 g/ml), morphology (“cup” or “dish” shaped in transmission electron microscopy), and certain enriched protein markers (tetraspanins, tumor susceptibility gene 101 (TSG101), heat shock proteins 70 (Hsp70)) [18]. The biogenesis of exosomes initiates from the internalization of membrane microdomains, which is the process for forming early endosomes (EEs). The EEs then migrate to multivesicular bodies (MVBs) and bud inwardly to form intraluminal vesicles (ILVs), which is the main progress for vesicles receiving their cargoes. Finally, after MVBs fuse with the cell membrane, exosomes are released from parental cells [19, 20]. The cargoes conveyed by exosomes contain proteins, lipids, and nucleic acids, and the loading of these cargoes is not random [21].

Different mechanisms are involved in sorting cargoes into exosomes. Membrane lipids of exosomes, such as different long-chain fatty acids, phosphatidylserine, and cholesterol, can accelerate the prioritized entry of simple lipids that are opposed to phospholipids [22]. Lipid raft domains on exosomal membrane may be associated with the types of proteins localized on membrane of exosomes [23]. However, the exact mechanism directing the composition of lipids to exosomes still remains unknown.

In the case of sorting proteins into exosomes, the endosomal-sorting complex required for transport (ESCRT) mechanism plays a critical role. ESCRT is a complex consisting of ESCRT-0, ESCRT-I, ESCRT-II, ESCRT-III, and associated proteins. The hepatocyte growth factor–regulated tyrosine kinase substrate (Hrs) Fab1p-YOTB-Vps27p-EEA1 (FYVE) domain of ESCRT-0 recognizes and interacts with phosphatidyl inositol 3-phosphate (PtdIns3P) of ubiquitinated proteins, and then the ubiquitinated proteins are recruited to the endosomal membrane. At the same time, ESCRT-0 recruits ESCRT-I with its Hrs presenilin-associated protein (PSAP) domain interacting with TSG101 of ESCRT-I. ESCRT-I then recruits ESCRT-II, which is the activator of ESCRT-III complex. ESCRT-III protein Snf7 activated by ESCRT-II recruits the adaptor protein ALG-2-interacting protein X (ALIX) to stabilize ESCRT-III, and promotes vesicle budding by forming oligomeric assemblies. When the assembly completes, ESCRT-III dissociates from the membrane with the assistant of ATPase vacuolar protein sorting protein 4 (Vps4) [21, 24]. In addition, there still exist ESCRT-independent mechanisms involved in sorting proteins into exosomes, since other post-translational modifications have also been found. For example, the acylation of the N-terminal domain promotes the protein to be packaged into exosomes [25].

Besides proteins and lipids, nucleic acids in exosomes have also been widely reported. However, the mechanism of sorting DNAs into exosomes still needs further investigation. Meanwhile, there have been some studies on the package of non-coding and coding RNAs into exosomes. For microRNA (miRNA) cases, ESCRT also plays an important role. Other ESCRT-independent mechanisms have at the same time been found, such as neutral sphingomyelinase 2 (nSMase2)-dependent pathway, heterogeneous nuclear ribonucleoprotein (hnRNP)-dependent pathway, post-transcriptional 3′ end modifications of miRNAs and RNA-induced silencing complex (miRISC)-related pathway: (a) nSMase2 is the rate-limiting enzyme of ceramides that are enriched in the plasma and exosomal membrane. Inhibiting the activity of nSMase2 does not affect the amount of miRNAs in parent cells, but decreases the quantity of miRNAs in exosomes, indicating ceramides are important for packaging miRNAs into exosomes [21, 26, 27]; (b) HnRNPA2B1 is a ubiquitous protein that can control the intracellular trafficking of specific mRNAs to distal sites in neurons. Sumoylated hnRNPA2B1 recognizes and binds specific motifs at the 3′ untranslated region (UTR) of miRNAs, and then transports miRNAs with these motifs into exosomes. Motifs involved in this process include GGAG, UGAG, CCCU, and UCCU localized at the 3′ end of miRNAs [28, 29]; (c) Studies about B cell-derived exosomal miRNAs suggest that miRNAs in B cell-derived exosomes own uridylated 3′ ends, while miRNAs from the parent cells share adenylated 3′ ends, reflecting that the 3′ end modification of miRNAs may be a conserved mechanism for sorting miRNAs into exosomes [30]; (d) Argonaute 2 (Ago2) is a key component of miRISC and knockdown of Ago2 downregulates the expression of miRNAs in exosomes derived from 293 T cells [31]. Besides that, miRISC has also been found to interact with MVBs directly, which indicates that the miRISC pathway is associated with loading of miRNAs into exosomes [32]. Besides miRNAs, long non-coding RNAs (lncRNAs) represent nearly 3% of RNAs in exosomes and can also be transferred into recipient cells. Studies on lncRNAs in TEXs demonstrate that exosomal lncRNAs are able to promote the invasion and metastasis of cancer by decreasing the apoptosis of cancer cells and facilitating the angiogenesis [33, 34]. Besides their effect on tumor cell biology, lncRNAs in TEXs can also regulate the development of immune cells and anti-tumor immune responses. For example, exosomal lncRNA growth arrest–specific 5 (GAS5) is capable of mediating the apoptosis of macrophage (Mϕ), hematopoiesis, and immune response [35, 36]. However, the cause of lncRNAs selectively being enriched in exosomes remains unclear. Similarly, although circular RNAs (circRNAs) are enriched and stable in exosomes, the mechanism for modulating the package of circRNAs into exosomes still needs to be identified [37, 38]. In addition to non-coding RNAs (ncRNAs), messenger RNAs (mRNAs) in exosomes also show selective enrichment. Loading of mRNAs into exosomes is directed by a conserved sequence of 25 nucleotides with a CTGCC core at the 3′ UTR of mRNAs [39].

**Fig. 1:**
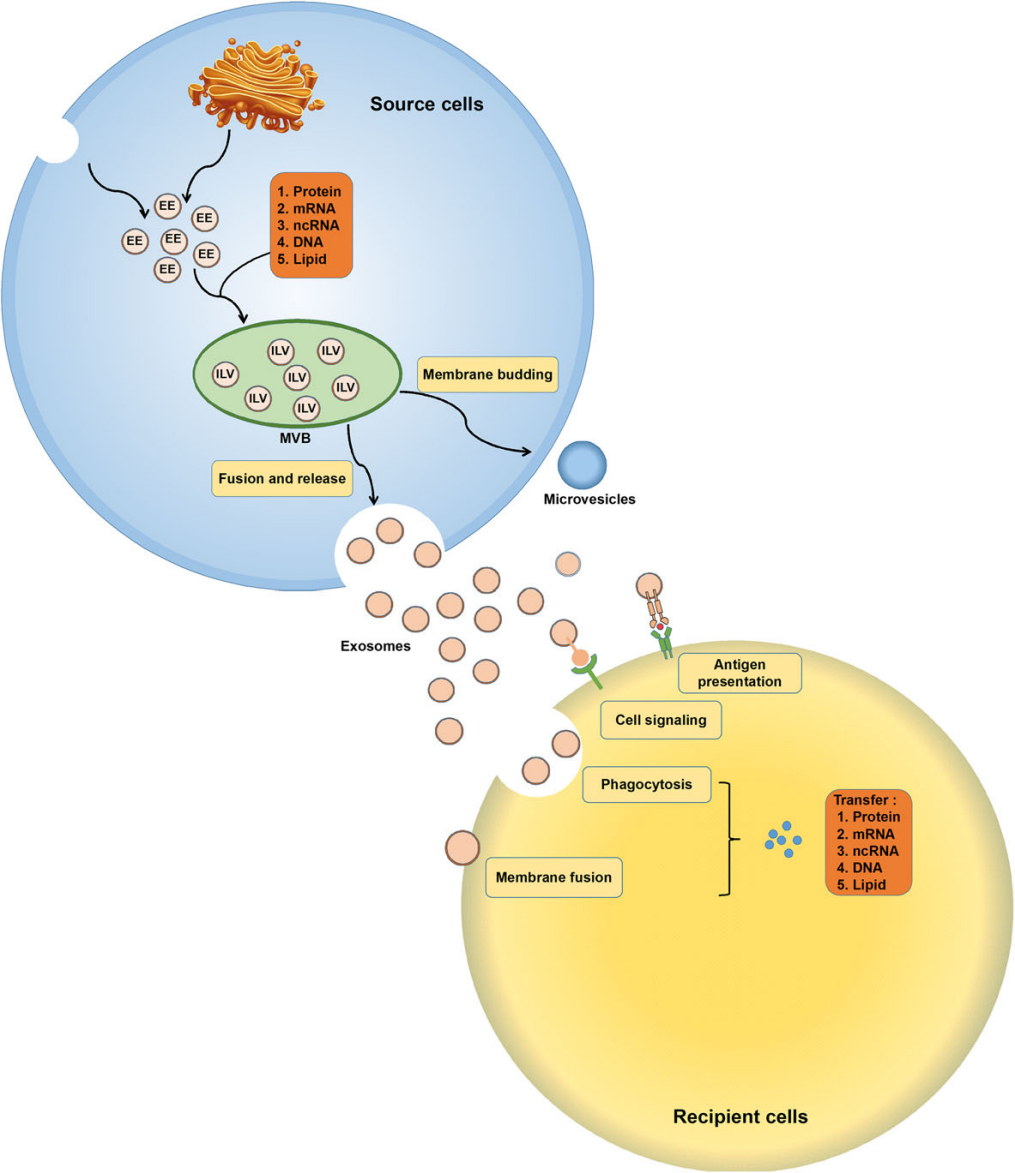
The formation and regulatory mechanism of exosomes. Exosome biogenesis initiates from the formation of EEs, which derive from the TGN and internalization of membrane microdomains. Then, EEs move into MVBs. During the inward budding of EEs into MVBs, vesicles load different cargoes and form ILVs. In this procedure, the loading of small plasma that contains nearly 100 proteins and 10000 nucleotides with proteins, coding and non-coding RNA, and DNA is a non-random process. Ras-related proteins regulate MVB movement towards cell membrane. MVBs fuse with the plasma membrane, and ILVs released to extracellular space are called exosomes. Exosomes received by recipient cells can be regarded as signalosomes for several biological processes. They can transfer both major histocompatibility complex (MHC) molecule and antigen, thereby involved in antigen presentation and immune regulation. Exosomes can also directly bind cell surface receptors and activate associated pathways. Additionally, exosomes can convey effectors including transcription factors, oncogenes, and infectious particles into recipient cells. Meanwhile, various nucleic acids are contained in extracellular vesicles and can be functionally delivered into recipient cells

Upon MVBs moving forward to fusing with the plasma membrane, ILVs are released to extracellular space as exosomes. Released exosomes can bind recipient cells relying on ligand-receptor interaction and activate associated signaling pathways to modulate the cell biology [18]. Besides that, the membrane of exosomes can directly fuse with the membrane of recipient cells, and then release the functional components inside. Another modality for exosome uptake is endocytosis that contains uptake induced by lipid rafts and caveolae, clathrin-dependent endocytosis, macropinocytosis, and phagocytosis. Once exosomes are internalized, contents of exosomes are transferred into recipient cells and induce the alteration of cell biology [17, 21]. The formation and regulatory mechanism for exosomes described above are shown in Fig. 1.

TEXs represent exosomes released by tumor cells, which are ubiquitously present in the tumor tissue and body fluids of cancer patients [40, 41]. The ratio of TEXs/normal cell–derived exosomes in the plasma of cancer patients is various, but generally, TEXs represent a majority of total exosomes in plasma of patients with advanced malignancies [42]. In TME, TEXs participate in intercellular cross-talk and transfer messages from the parent tumor cells to recipient cells, including MDSCs. TEXs are able to modulate autocrine, juxtacrine, and paracrine signaling pathways that are essential for the survival of cancer cells [43]. Notably, the paracrine activity of TEXs is not only limited to the tumor site because TEXs can circulate and convey information to tissues and cells distant from the tumor. Current studies have demonstrated that TEXs have the capacity of promoting the activation, expansion, and immunosuppressive function of MDSCs [44].

## MDSCs

**Fig. 2:**
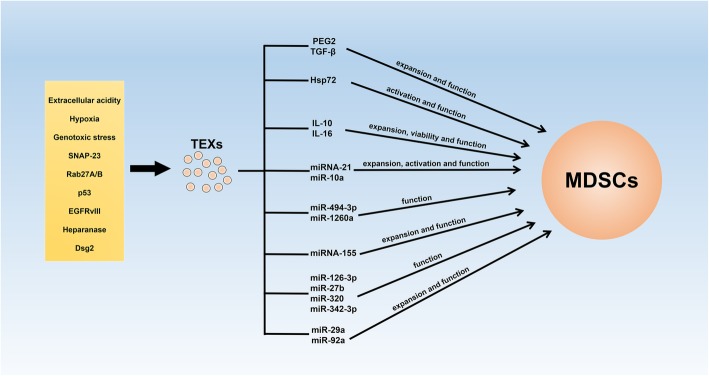
TEXs participate in the cell-cell communication between tumor cells and MDSCs. Environmental conditions, such as extracellular acidity, hypoxia, genotoxic stress, and associated proteins in TME are capable of contributing to the formation and release of TEXs. Released TEXs are able to enhance the activation, expansion, and immunosuppression of MDSCs by conveying functional cargoes

MDSCs are a population of heterogeneous cells that mainly consist of immature myeloid cells (IMCs). Under physiological conditions, these IMCs come into being in the bone marrow (BM) and differentiate into mature monocytes, dendritic cells (DCs), and granulocytes immediately [45]. Nevertheless, in a pathological environment, especially cancer, the differentiation and maturation of these IMCs are blocked, which leads to the expansion of MDSCs in vivo [10, 46]. In TME, the expansion and immunosuppression of MDSCs are induced by a variety of molecules that are produced by tumor cells, stromal cells, and activated immune cells. These molecules can be divided into two groups: (a) The first group is crucial for the expansion of MDSCs. Granulocyte-macrophage colony–stimulating factor (GM-CSF), granulocyte colony–stimulating factor (G-CSF), macrophage colony–stimulating factor (M-CSF), stem cell factor (SCF), vascular endothelial growth factor (VEGF), and polyunsaturated fatty acids are included in this group [47, 48]. Transcriptional factors/regulators, including signal transducers and activators of transcription 3 (STAT3), interferon regulatory factor (IRF8), CCAAT/enhancer-binding protein-β (C/EBP-β), and NOTCH, have major roles in pathways activated by these molecules [49]; (b) The second group consist of inflammatory cytokines and damage-associated molecular patterns (DAMPs) that are responsible for MDSC activation. Interferon-γ (IFN-γ), interleukin-1β (IL-1β), IL-4, IL-6, IL-13, tumor necrosis factor (TNF), and high-mobility group box 1 (HMGB1) are included in this group. These factors mainly signal via nuclear factor-kappa B (NF-κB), STAT1, and STAT6 [49]. Besides that, molecules released from tumor cells and stromal cells, oxidative phosphorylation, and glycolysis are also closely associated with the immunosuppressive function of tumor-infiltrated MDSCs. Currently, endoplasmic reticulum (ER) stress is emerging as a crucial regulator of the activation and suppressive function of MDSCs. ER stress enhances the immunosuppression of MDSCs by promoting the expression of arginase 1 (Arg1), nitric oxide synthase 2 (NOS2), and NADPH oxidase 2 (NOX2). Furthermore, ER stress can accelerate the apoptosis of MDSCs in the spleen through activating TNF-related apoptosis-induced ligand receptor 2 and caspase-8, and facilitating the accumulation of MDSCs in BM [50, 51]. Additionally, recent studies demonstrate that exosomes released by cancer cells are able to modulate the activation, expansion, and immunosuppression of MDSCs [5, 6]. To confirm the accurate mechanism for TEXs regulating MDSCs, functional components in TEXs have also been identified [17]. However, although cargoes conveyed by exosomes are various, current studies on functional components in TEXs that mediate the cell biology of MDSCs are mainly focused on proteins and miRNAs, which are two focuses that we will discuss in this review. The influences of TEXs on the cell biology of MDSCs are shown in Fig. 2.

Due to the heterogeneity, the phenotype of MDSCs is complicated. In mice, MDSCs are characterized as CD11b^+^Gr1^+^ cells. Based on the different expression of two Gr1 subsets, MDSCs can be further divided into CD11b^+^Ly6G^+^Ly6C^low^ polymorphonuclear myeloid-derived suppressor cells (PMN-MDSCs) and CD11b^+^Ly6G^-^Ly6C^high^ monocytic myeloid-derived suppressor cells (M-MDSCs) [52, 53]. In humans, PMN-MDSCs are defined as CD11b^+^CD14^-^CD15^+^CD33^+^ which share the same phenotype with neutrophils [10]. Recently, lectin-type oxidized LDL receptor 1 (LOX-1) is identified to better distinguish human PMN-MDSCs from neutrophils. It is reported that LOX-1^+^ PMN-MDSCs represent nearly 10% of neutrophils in the peripheral blood of cancer patients, and up to 40% of neutrophils in tumor tissues [10, 54]. For human M-MDSCs, relying on the different expression of human leukocyte antigen DR (HLA-DR), CD11b^+^CD14^+^CD15^-^CD33^+^HLA-DR^-/low^ M-MDSCs can be easily distinguished from HLA-DR^+^ monocytes [52, 55]. Besides the phenotype, MDSCs can also be distinguished from neutrophils and monocytes by gene- and protein-expressing profiles. Compared to the neutrophils, there are higher levels of genes involved in cell cycle, autophagy, the cAMP-response element-binding protein (CREB) pathway, and G protein signaling in PMN-MDSCs [56]. Data from sequencing analysis indicates that increased ER stress, upregulation of mammalian target of rapamycin (mTOR) signaling, the mitogen-activated protein kinase (MAPK) pathway, and IFN-γ-regulated pathway exist in human PMN-MDSCs compared with mature neutrophils [54]. Additionally, analysis of proteomics demonstrates that MDSCs constitute a distinct myeloid population characterized by a “kinase signature” and well-defined interactomes [57, 58].

The most important feature of MDSCs is inhibiting immune responses and promoting the tumor progression. MDSCs produce high levels of suppressive molecules, such as Arg1, reactive oxygen species (ROS), inducible nitric oxide synthase (iNOS), and prostaglandin E2 (PGE2), to directly suppress effector T cell–induced anti-tumor immune response [45, 59–61]. MDSCs can also suppress the immune response by inducing regulatory T cells (Tregs), promoting the differentiation of T helper cell 17 (Th17), facilitating macrophage developing into M2 phenotype, and differentiating into tumor-associated macrophages (TAMs) [45, 62, 63]. Additionally, recent studies suggest that MDSC-exosomes are capable of mediating the immune response and development of target immune cells [17]. Notably, exosomes from MDSCs can directly accelerate the proliferation and metastasis of tumor cells by conveying miR-126a, which indicates a new regulatory mechanism for MDSCs on tumor cells [64]. MDSC-induced immunosuppression facilitates the tumor progression by promoting the epithelial-mesenchymal transition (EMT), accelerating the immune escape and enhancing the formation of metastatic lesions [10].

## TME

TME consists of surrounding blood vessels, ECM, fibroblasts, lymphocytes, signaling molecules, immune cells, and bone marrow-derived inflammatory cells. It is the cellular environment in which a tumor exists [65]. The interaction between stromal cells and tumor cells influences the initiation and progression of cancer. Tumor cells accelerate the formation of TME through promoting tumor angiogenesis, releasing extracellular molecules, and inducing immune tolerance. Meanwhile, TME is able to influence the growth and evolution of cancer cells, and contribute to tumor heterogeneity [7, 66].

A mass of stromal cell types in TME contribute to the formation of tumorigenic primary niches [67]. The immune system is immediately activated to eliminate tumor cells post these cells circumvent cell-intrinsic mechanisms of apoptosis. In this procedure, tumor-specific antigens are recognized by adaptive immune cells that lead to the destruction of tumor cells. At this moment, macrophages and fibroblasts in TME also suppress the proliferation of tumor cells. However, these cells will later be educated by tumor cells and become pro-tumorigenic [68, 69]. As tumor expands, immune cells with suppressive activity, including MDSCs, Tregs, and TAMs, infiltrate into tumor tissues and induce immunosuppression to promote tumor escape via disrupting antigen presentation by DCs, inhibiting T and B cell proliferation and activation, and suppressing natural killer cell (NK) cytotoxicity [70–73]. Besides that, carcinoma-associated fibroblasts (CAFs) activated by molecules from tumor cells can secrete ECM proteins and basement membrane components, regulate immune responses, and contribute to deregulated homeostasis [74–76]. CAFs are also a key source of VEGF that supports angiogenesis during tumor growth [76]. In addition to cellular contributions, several extracellular properties in TME, including low oxygen tension, high interstitial fluid pressure, and changes in specific components of the ECM, also contribute to tumor progression [7].

TME also promotes the metastatic dissemination and colonization of cancer at secondary sites. Cells including MDSCs, TAMs, and platelets facilitate the EMT at primary sites, leading to the separation of tumor cells from neighboring epithelial cells and promoting tumor cells acquiring a mobile and invasive phenotype [71, 77, 78]. In this process, transforming growth factor-β (TGF-β) produced by tumor stroma acts as a key regulator and is involved in a paracrine signaling loop with tumor cells. Meanwhile, cells with suppressive activity, such as TAMs, CAFs, and MDSCs, also tend to cluster at the leading edge of the primary tumor to inhibit DC differentiation [7]. During endosmose of tumor cells into the circulation, macrophages are localized to perivascular areas in tumor and assist tumor cells to pass through vessel barriers [79, 80]. In the peripheral circulation, platelets, and proteins of the coagulation system enhance the survival of tumor cells through preventing them from being recognized by cytotoxic immune cells. Tumor cells protected by platelets in the circulation migrate to the extravasation site, and platelets then bind to areas of vascular retraction to help tumor cells enter into secondary organs [81, 82]. In secondary organs, the expression of fibronectin is upregulated to serve as a docking site for hematopoietic progenitor cells (HPCs) and tumor cells, and cells with suppressive activity such as MDSCs promote the metastasis of cancer by creating a niche that is permissive to tumor colonization [7]. It has recently been proven that TEXs are able to modulate the communication between primary and metastatic lesions of cancer by conveying cargoes into tumor cells, immune cells, and stromal cells. Exosomal components are capable of directing organ tropism, modulating immune evasion, and supporting the EMT. Moreover, TEXs are predictive for metastasis and patients’ outcome [19, 20, 83]. Thus, TEXs are emerging more and more important for intercellular communication in TME. Besides that, environmental conditions, such as extracellular acidity, hypoxia, genotoxic stress, and associated proteins in TME, are capable of contributing to the formation and release of TEXs, which we will elaborate in this review and are shown in Fig. 2.

## TME promotes the formation and release of TEXs

A large number of studies suppose that cancer cells release much more exosomes compared with non-malignant cells. It makes exosomes become attractive targets for anti-tumor therapy [20]. Indeed, ESCRT components as well as syntenin and heparanase have been found to be over-expressed in various tumors [84, 85]. Apart from cell-intrinsic mechanisms, tumor microenvironmental conditions, such as hypoxia, are capable of contributing to the formation and release of TEXs [86, 87]. In the following chapter, we summarized the most recent researches revealing the regulation of TME on TEXs in cancer. All the tumor microenvironmental conditions that are capable of contributing to the formation and release of TEXs, and the accurate regulatory mechanisms are listed in Table 1.

**Table 1:** TME promotes the formation and release of TEXs

Modulator	Type of cancer	Observation	Refs
Extracellular acidity	Melanoma	Human metastatic melanoma cells release more exosomes under an acidic condition. Ganglioside GM3 and caveolin-1 are enriched in TEXs released at low pH.	[88]
	Prostate cancer	Acidity of TME facilitates the release of TEXs expressing PSA and CD81. Extracellular acidity leads to the spill-over of TEXs into the peripheral blood of patients with prostate cancer.	[89]
Hypoxia	Lung cancer	Lung cancer cells produce more miR-23a-contained exosomes under hypoxic condition. Exosomal miR-23a can enhance the angiogenesis, vascular permeability, and transendothelial migration of lung cancer.	[90]
	Ovarian cancer	Hypoxia significantly increases the release of TEXs in ovarian cancer by reducing endolysosomal fusion and increasing the expression of TFEB. Additionally, hypoxic conditions induce the upregulation of Rab27a and downregulation of Rab7 by activating STAT3 to promote the release of TEXs.	[91]
	Bladder cancer	LncRNA-UCA1 is enriched in hypoxic bladder cancer cell-derived TEXs, and hypoxic exosomal lncRNA-UCA1 can promote tumor growth and progression though accelerating EMT.	[92]
Genotoxic stress	Multiple myeloma	Genotoxic stress facilitates the release of exosomes from MM cells. MM cell-derived exosomes are able to promote IFN-γ production of NK cells in TME by activating NF-κB pathway in a TLR2/Hsp70-dependent manner.	[93]
SNAP-23	Lung cancer	Phosphorylated PKM2 form a dimer structure with low pyruvate kinase activity but high protein kinase activity. It then associates with SNAP-23 near cells' membranes, leading to SNAP-23 phosphorylation at Ser95 and upregulation of TEXs release	[94, 95]
Rab27A Rab27B	Bladder cancer /Cervical cancer /Breast cancer /Melanoma /Lung adenocarcinoma	Rab27A regulates docking and membrane fusion of MVEs, whereas Rab27B participates in the transfer of membranes from the TGN to MVEs. Rab27A/B-induced exosome secretion decreases the expression of tumor-suppressive microRNAs.	[96, 97]
p53	Lung cancer	TSAP6 and maspin induced by p53 play a key role in the exosome-mediated secretion. The secreted proteins may be involved in cell-cell communication.	[98]
	Colorectal cancer	Knockdown of TP53 which is encoding gene of p53 protein induces colorectal cancer cells to produce exosomes with reduced sizes in a HGS-dependent manner.	[99]
	Gastric cancer	p53^−/−^ MSC exosomes deliver UBR2 to target cells and promote gastric cancer growth and metastasis by regulating Wnt/β-catenin pathway.	[100]
EGFRvIII	Glioma	EGFRvIII changes the expressing profile of exosome-associated proteins and their protein compositions in GBM. TEXs from EGFRvIII expressing glioma cells are enriched with focal adhesion related proteins to promote the invasion of cancer cells.	[101]
Heparanase	Lyeloma/lymphoblastoid/breast cancer	Heparanase drives exosome secretion, alters exosome composition, and facilitates production of exosomes that impact both tumor and host cell behavior.	[102]
	Mammary ductal carcinoma	Heparanase promotes endosomal membrane budding and modulates the biogenesis of exosomes by activating the syndecan-syntenin-ALIX pathway.	[103, 104]
Dsg2	Lung cancer	C-terminal fragment of Dsg2 enhances the release of TEXs and promotes the package of EGFR and c-Src into TEXs.	[105]

Lists the tumor microenvironmental conditions discussed in this review which are capable of contributing to the formation and release of TEXs

### Extracellular acidity

Extracellular acidity due to lactic acid and H^+^ accumulation is a common characterization of TME. It is indicated that extracellular acidity of the TME increases the release of TEXs. In the study by Parolini I et al., human metastatic melanoma cells, which produce constant exosomes and are able to sustain a low pH condition without showing cytotoxicity, were found to release more exosomes under an acidic condition (pH = 6.0) than that under a buffered condition (pH = 7.4). The following membrane biophysical analysis suggested that monosialodihexosylganglioside (GM3) content, which was likely responsible for the increased fusion efficiency, was enriched in exosomes released at low pH. Furthermore, exosomes secreted by melanoma cells in an acidic condition could deliver caveolin-1 that is a protein involved in melanoma progression [88]. In prostate cancer, the acidity of TME performs as a selective pressure, which facilitates the release of TEXs expressing prostate-specific antigen (PSA) and CD81. Besides that, extracellular acidity leads to the spill-over of TEXs into the peripheral blood of prostate cancer patients, indicating that TEXs may act as a non-invasive clinical tool for the screening and early diagnosis of prostate cancer [89].

### Hypoxia

Hypoxia is another feature playing a critical role in TME and in the evolution of malignant cells. It was found that compared to parental cells under normoxic condition, lung cancer cells produce more exosomes under hypoxic conditions. miR-23a expression is significantly upregulated in exosomes released under hypoxic conditions in lung cancer cells. Exosomal miR-23a accelerates the accumulation of hypoxia-inducible factor-1α (HIF-1α) in endothelial cells through targeting prolyl hydroxylase 1/2 (PHD1/2). Based on this regulation of exosomal miR-23a on HIF-1α, exosomes derived from lung cancer cells under hypoxic condition enhance the angiogenesis of tumor. Additionally, exosomal miR-23a also suppresses the expression of tight junction protein zona occludens protein 1 (ZO-1), thereby increasing the vascular permeability and cancer transendothelial migration [90]. In ovarian cancer, hypoxia significantly increases the release of TEXs by reducing endolysosomal fusion and increasing the expression of transcription factor EB (TFEB) that can favor the lysosome docking. It is also implied that hypoxic conditions induce the upregulation of Rab27a and downregulation of Rab7 by activating STAT3 to promote the release of TEXs from ovarian cancer cells with an altered lysosomal phenotype. Moreover, oncogenic proteins conveyed by TEXs promote the tumor progression, the chemo-resistance, and reprogramming of fallopian tube secretory epithelial cells (FTSECs) [91]. Meanwhile, hypoxia enhancing TEX release has also been revealed in breast cancer, bladder cancer, and prostate cancer [86, 92, 106]. Besides promoting the release of TEXs, hypoxia in TME can also modulate the loading of cargoes into TEXs. It is demonstrated that lncRNA urothelial carcinoma-associated 1 (UCA1) is enriched in hypoxic bladder cancer cell-derived exosomes, and hypoxic exosomal lncRNA UCA1 promotes tumor growth and progression though accelerating EMT [92].

### Genotoxic stress

Anti-cancer chemotherapy that enhances the immunogenic potential of malignant cells is mainly based on the establishment of immunogenic cell death (ICD) and the release of DAMPs. It is demonstrated that genotoxic stress is induced by melphalan, which is a genotoxic agent used in multiple myeloma (MM) therapy, facilitating the release of exosomes from MM cells. MM cell–derived exosomes are able to promote IFN-γ production of NK cells in TME by activating the NF-κB pathway in a Toll-like receptor 2 (TLR2)/Hsp 70-dependent manner but not the cytotoxic activity. Moreover, Hsp70^+^ exosomes are primarily found in the BM of MM patients, which implies their crucial immunomodulatory actions in TME [93].

### Synaptosome-associated protein 23

Similar to other cell types, tumor cells in TME employ the soluble N-ethylmaleimide-sensitive fusion factor attachment protein receptor (SNARE) complex to release exosomes. The SNARE complex is comprised of v-SNARE on membrane of budding vesicles and t-SNARE on the cells^,^ membrane, which enable the subsequent fusion of the two membranes thereby mediating exosome release. In tumor cells, syntaxin-4 and synaptosome-associated protein 23 (SNAP-23) serve as t-SNARE, when vesicle-associated membrane protein-2 (VAMP-2) and VAMP-8 represent v-SNARE. Phosphorylated SNAP-23 is the phosphorylated substrate of pyruvate kinase type M2 (PKM2) in tumor cells that can directly promote the release of exosomes. During exosome secretion, phosphorylated PKM2 forms a dimer structure with low pyruvate kinase activity but high protein kinase activity. It then associates with SNAP-23 near the cell membrane, leading to SNAP-23 phosphorylation at Serine 95 and upregulation of TEX release [94, 95].

### Rab27A and Rab27B

Rab27A and Rab27B are small guanosine triphosphate (GTPases) (20-25 kDa) that belong to the Rab protein family. Both Rab27A and Rab27B have been reported to promote the proliferation, enhance the invasion, and increase the chemo-resistance of cancer [107]. Meanwhile, Rab27A and Rab27B are important molecules regulating exosome trafficking. Rab27A and Rab27B can perform as molecular switches that oscillate between the GTP-bound active form and guanosine diphosphate (GDP)-bound inactive form to regulate the secretion of exosomes. In the active form, Rab27 recruits effector proteins and coordinates the vesicle trafficking process, including vesicle sorting, uncoating, motility, tethering, and fusion [108]. Although Rab27A and Rab27B share 71% amino acid sequence identity [109], their roles in the exosome pathway are different. Rab27A regulates docking and membrane fusion of multivesicular endosomes (MVEs), whereas Rab27B participates in the transfer of membranes from the trans-Golgi network (TGN) to MVEs. Knockdown of Rab27A or Rab27B reduces exosome release in different cancer cell types, including bladder cancer cells, cervical cancer cells, breast cancer cells, melanoma cells, and lung adenocarcinoma cells. It has also been identified that there exist 11 Rab27-specific effectors that are crucial for determining the efficiency and specificity of Rab27-mediated exocytosis [96]. Additionally, Rab27A/B-induced exosome secretion decreases the expression of tumor-suppressive microRNAs, including miR-23b and miR-921, leading to cancer growth and metastasis [97]. All these indicate the crucial role of Rab27A and Rab27B in regulating exosome trafficking.

### p53

A variety of stress signals such as genotoxic stress and hypoxia activate the p53 associated pathway. After DNA damage, the p53 protein is activated to become a transcription factor to modulate the transcription of target genes. The research by Yu X et al. found that a set of proteins encoded by genes that are not transcriptional targets of p53 increased in the culture medium of lung cancer cells after p53 activation and that these proteins were secreted into the medium via exosomes. Furthermore, evidence was presented that p53 transcribed the transmembrane protein tumor suppressor-activated pathway 6 (TSAP6) gene whose product was sufficient to induce the secretion of exosomes [98]. Another study revealed that knockdown of TP53, which is the encoding gene for p53 protein, could induce colorectal cancer cells to produce exosomes with reduced sizes in a hepatocyte growth factor–regulated tyrosine kinase substrate (HGS)-dependent manner [99]. Meanwhile, p53 deficient mesenchymal stem cells (MSC) produce more exosomes that are enriched with ubiquitin-protein ligase E3 component n-recognin 2 (UBR2) and can promote gastric cancer progression via Wnt/β-catenin pathway [100].

### Epidermal growth factor receptor vIII

Glioblastoma multiforme (GBM) is a highly aggressive brain tumor associated with rapid cell proliferation and therapeutic resistance. The active epidermal growth factor receptor vIII (EGFRvIII) is commonly related to GBM progression and contributes to the aggressive feature of tumor cells as well as alterations in TME. A previous study has indicated that exosomal EGFRvIII can fuse with plasma membrane of GBM cancer cells lacking EGFRvIII, leading to the activation of MAPK and Akt pathways, and inducing changes in the expression of EGFRvIII-regulated genes and the morphological transformation [110]. A recent work by Choi D et al. revealed that EGFRvIII changed the expressing profile of exosome-associated proteins and their protein compositions in GBM. Exosomes from EGFRvIII expressing glioma cells were enriched with focal adhesion-related proteins, such as CD44 and CD151, to promote the invasion of cancer cells. They also found that levels of homophilic adhesion molecules were enhanced and that increased homologous exosomes were internalized by EGFRvIII-positive glioma cells. These results suggest that oncogenic EGFRvIII induced by TME reprograms the proteome and uptake of GBM-related exosomes [101].

### Heparanase

Heparanase is the sole heparan sulfate degrading endoglycosidase and is increased in many tumors. Heparanase can enhance the growth, metastasis, angiogenesis, and inflammation of tumor. In a recent study, heparanase was found to facilitate the secretion of exosomes and alter both their composition and biological function. When cancer cells were exposed to exogenous heparanase or the expression of heparanase was promoted, the release of TEXs increased dramatically. At the same time, exosomal protein cargoes were also altered by over-expressed heparanase, with increased package of proteins associated with an aggressive tumor phenotype. Meanwhile, TEXs secreted by cells over-expressing heparanase altered the cell biology of both tumor cells and host cells [102]. Moreover, heparanase promotes the endosomal membrane budding and modulates the biogenesis of exosomes by activating the syndecan-syntenin-ALIX pathway [103, 104].

### Desmoglein 2

Desmoglein 2 (Dsg2) belonging to desmosomal cadherins is expressed in all epithelial-derived tissues. The C-terminal fragment of Dsg2 is a desmosomal cadherin over-expressed in malignancies. Overmiller AM et al. found that TEXs from squamous cell carcinoma (SCC) were enriched with the C-terminal fragment of Dsg2 for the first time. Upregulation of Dsg2 C-terminal fragment enhanced the release of TEXs and promoted the package of epidermal growth factor receptor (EGFR) and c-Src into TEXs. Downregulating ectodomain shedding of Dsg2 led to a reduced release of TEXs and the accumulation of full-length Dsg2 in TEXs. TEXs enriched with the C-terminal fragment of Dsg2 could be internalized by CD90^+^ fibroblasts and promote the proliferation of fibroblast cells through activating extracellular regulated protein kinase (Erk) 1/2 and Akt pathways. Besides that, Dsg2 C-terminal fragment and EGFR were abundant in TEXs isolated from sera of SCC patients. All these indicate that Dsg2 can modulate the secretion of TEXs, which are critical for tumor progression [105].

## TEXs induce the expansion and enhance the immunosuppression of MDSCs

MDSCs play a crucial role in the immune escape of cancer. In TME, cytokines, which are produced by tumor cells, stromal cells, and activated immune cells, induce the activation, expansion, and immunosuppression of MDSCs. The downstream signals mainly include the janus kinase (JAK)-STAT pathway and the myeloid differentiation factor 88 (MyD88)-NF-kB pathway [10, 111, 112]. Currently, exosomes released by various tumor cells have been demonstrated to play a crucial role in the expansion, survival, and immunosuppression of MDSCs. A research by Valenti R et al. found that melanoma-derived exosomes promoted myeloid cells differentiating into TGF-β-secreting CD14^+^HLA-DR^−^ cells, while inhibiting the differentiation of myeloid cells to DCs [5]. Functional analysis suggested that TEX-induced MDSCs were capable of polarizing monocyte to M2 phenotype expressing a high level of CD163, along with the formation of tumor-promoting microenvironment and accelerated Th2 immune response [6]. TEXs are also able to promote the survival of MDSCs by enhancing the expression of anti-apoptotic protein B cell lymphoma-extra large (Bcl-xL) and activating STAT1/3 pathway [113]. Moreover, TEXs boost the production of suppressive molecules from MDSCs and enhance their suppressive activity in tumor models [114]. All these emphasize the importance of TEXs in the cell biology of MDSCs, and the exact regulatory mechanisms for TEXs on MDSCs have also been revealed gradually. In the following chapter, we discuss the influence of TEXs on MDSCs biology by conveying different functional components that are mainly proteins and nucleic acids. The detailed information on proteins and miRNAs in TEXs, which can modulate the expansion and function of MDSCs, and their specific regulatory mechanisms are contained in Table 2.

**Table 2:** Proteins/miRNAs in TEXs modulate the development and function of MDSCs

Functional components	Type of cancer	Observation	Refs
Proteins			
PGE2	Mammary carcinoma	TEXs with abundant PGE2 and TGF-β enhance the expansion and immunosuppression of MDSCs depending on MyD88 pathway by increasing the production of IL-6 and VEGF.	[114, 115]
TGF-β			
Hsp72	Colon carcinoma /mammary carcinoma/lymphoma	Hsp72 expressed on the membrane of exosomes from tumor cells triggers STAT3 activation in MDSCs depending on the TLR2/MyD88 pathway through autocrine of IL-6.	[116]
IL-10	Multiple myeloma	Exosomal IL-10 and IL-16 from MM cells increase the accumulation and enhance the suppressive function of BM MDSCs by activating STAT3 pathway. MM exosomes can also reduce the survival of PMN-MDSCs, while increasing the survival of M-MDSCs.	[113, 117]
IL-16			
miRNAs			
miRNA-21	Hypoxia-induced glioma	miRNA-21 and miR-10a in exosomes from hypoxia-induced glioma promote the expansion and immunosuppression of MDSCs by targeting PTEN and RORα.	[118]
miR-10a			
	Oral squamous cell carcinoma	Hypoxic TEXs enhance the suppressive function of MDSCs and attenuate γδ T-cell activity in a miR-21/PTEN/PD-L1-axis-dependent manner.	[119]
miR-494-3p	Pancreatic ductal adenocarcinoma	PDAC-exosomes create an immunosuppressive myeloid cell background by increasing calcium fluxes through the transfer of SMAD4-related differentially expressed miR-1260a and miR-494-3p.	[120]
miR-1260a			
miRNA-155	B- cell-derived chronic lymphocytic leukemia	High level of exosomal miRNA-155 from CLL cells can be uptaken by monocytes and induce IDO expressing MDSCs through STAT1 pathway.	[121]
miR-126-3p	Glioma/lung cancer	MDSCs internalizing TEXs display enhanced expression of suppressive molecules and differing miRNA profiles including miR-126-3p, miR-27b, miR-320, and miR-342-3p.	[122]
miR-27b			
miR-320			
miR-342-3p			
miR-29a	Glioma	TEXs from glioma mediate the expansion and function of myeloid-derived suppressor cells through microRNA-29a/Hbp1 and microRNA-92a/Prkar1a pathways.	[123]
miR-92a			

Contains the detailed information of proteins and miRNAs in TEXs discussed in the review, which can modulate the development and function of MDSCs

## TEX-proteins involved in regulating the expansion and immunosuppression of MDSCs

### PGE2 and TGF-β

TEX-protein inducing MDSC expansion has been revealed for several years. A study by Xiang X et al. firstly indicated exosomes from tumor cells could be taken up by myeloid cells from the bone marrow, and myeloid cells taking up TEXs showed typical phenotypic and functional characteristics of MDSCs. TEXs could significantly induce the accumulation of MDSCs expressing cyclo-oxygen-ase 2 (Cox2), IL-6, VEGF, and Arg1, and promote the tumor progression. Their following findings moved forward to demonstrating that TEXs induced MDSCs from myeloid cells by conveying PGE2 and TGF-β molecules. Blockade of exosomal PGE2 and TGF-β abolished the induction of MDSCs by TEXs and downregulated MDSC-mediated immunosuppression. Moreover, TME modulated the TEX-induced MDSCs by accelerating the package of PGE2 and TGF-β into exosomes [114]. It was also demonstrated that MyD88, but not TRAF-interacting protein (TRIP) adaptor molecule, was responsible for TEX-mediated expansion of MDSCs [115]. In a recent study, they compared the biological effects of exosomes derived from in vitro cultured B16 tumor cells (named as C-exosomes for culture exosomes) and exosomes derived from in vivo grown B16 tumor (named as P-exosomes for primary exosomes). It was supposed that P-exosomes induced TLR2-independent MDSCs activation and expansion, whereas C-exosomes activated and expanded MDSCs relying on a TLR2-dependent manner [124]. All these findings promote the development of specific targetable therapeutic strategies of eliminating MDSC-induced immunosuppression and enhancing host anti-tumor immunotherapy efficacy.

### Hsp72

In the research by Chalmin F et al., Hsp72 expressed on the membrane of TEXs from murine colon carcinoma, mammary carcinoma, and lymphoma was shown to interact with MDSCs and modulate the immunosuppression of MDSCs by activating STAT3. At the same time, tumor-derived soluble factors were responsible for MDSC expansion via activating Erk pathway. Hsp72 on the membrane of TEXs triggered STAT3 activation in MDSCs, depending on a TLR2/MyD88 manner through autocrine production of IL-6. Importantly, dimethyl amiloride promoted the anti-tumor efficacy of the chemotherapeutic drug cyclophosphamide by reducing TEX production in murine models. TEXs from human tumor cells could also activate human MDSCs and triggered their suppressive function relying on an Hsp72/TLR2-dependent manner. Furthermore, MDSCs from cancer patients treated with amiloride, a drug used to treat high blood pressure that also inhibits exosome formation, exhibited reduced suppressive function. Collectively, these findings show that Hsp72 expressed on the surface of TEXs promotes the tumor escape by enhancing the immunosuppression of MDSCs [116].

However, different from the results of Xiang X et al., Chalmin F et al. showed that there was no detectable PGE2 in TEXs. They also identified that the activation of STAT3 by TEXs accounted for MDSC activation, whereas tumor-derived soluble factors were responsible for the expansion. For example, tumors in TLR2^−/−^ mice could induce the expansion of MDSCs, but not their activation [125]. The possible reason causing this difference may be that secreted PGE2 is packaged into exosomes outside host cells before being taken up by recipient cells.

### IL-10 and IL-16

Besides TEXs from solid tumors, exosomes from hematological malignancy can also enhance the suppressive capacity of MDSCs. Exosomes from MM are supposed to establish a bone marrow microenvironment by enhancing the angiogenesis and immunosuppression. It is identified that exosomes from MM cells can enhance the accumulation and viability of MDSCs in both murine models and MM patients by activating STAT3 pathway. MM exosomes also induce changes in MDSC subpopulations, which inhibit the survival of PMN-MDSCs and prolong the survival of M-MDSCs. MM exosomes significantly upregulate the expression of iNOS in MDSCs. In a further study identifying functional components in MM exosomes, exosomal IL-10 and IL-16 are found to be involved in the regulation to MDSCs. Meanwhile, exosomes from bone marrow stromal cells (BMSCs) are demonstrated to be taken up by MM MDSCs in MM, and mainly promote the survival of M-MDSCs. Moreover, BMSC-exosomes can also enhance the immunosuppression of MDSCs through activating STAT1/3, and increase the expression of Bcl-xL and myeloid cell leukemia-1 (Mcl-1) in MDSCs [113, 117].

## TEX-nucleic acids involved in regulating the expansion and immunosuppression of MDSCs

TEXs enable the direct transfer of nucleic acids that were ignored to be involved in cell-cell communication, particularly RNAs [33, 126, 127]. Besides that, the work by Ridder K et al. recently demonstrated that MDSCs were principal recipient cells for TEX**-**nucleic acids [122]. Currently, studies on the regulation of exosomal nucleic acids on MDSCs are mainly about exosomal miRNAs that account for 76.2% of total RNAs in exosomes, while the effect of exosomal lncRNAs and mRNAs are rarely reported [17, 128]. Thus, in this review, we focus on the effect of exosomal miRNAs on the cell biology of MDSCs.

### miRNA-21 and miR-10a

It was recently indicated that exosomes from hypoxia-induced glioma could promote BM cells differentiating into CD11b^+^Gr1^+^ MDSCs and enhance their immunosuppression by inducing the production of suppressive molecules, such as TGF-β, ROS, NO, and IL-10. In order to further confirm whether hypoxia-induced glioma exosomes (H-GDEs) regulated the accumulation and function of MDSCs through conveying miRNAs, miRNA expressing profile in H-GDEs was analyzed. 17 of the 20 highest expressed miRNAs were transfected into mouse BM cells to estimate their effect on MDSC expansion. In these miRNAs, the hypoxia-inducible expression of miR-10a and miR-21 in TEXs enhanced TEX-induced MDSC expansion and activation by targeting RAR-related orphan receptor alpha (RORα) and phosphatase and tensin homolog (PTEN). Mice inoculated with miR-10a or miR-21 knockout glioma cells generated fewer MDSCs than those inoculated with normal glioma cells [118]. In oral squamous cell carcinoma (OSCC), hypoxic TEXs enhance the suppressive function of MDSCs and attenuate γδ T-cell activity in a miR-21/PTEN/PD-L1-axis-dependent manner. Exogenous miR-21 transferred by hypoxic TEXs downregulates PTEN level in MDSCs and increases the expression of PD-L1, finally inducing the immunosuppressive activity of MDSCs [119].

### miR-1260a and miR-494-3p

In pancreatic ductal adenocarcinoma (PDAC), PDAC-exosomes are found to alter the phenotype of myeloid cells from DCs to M-MDSCs, by increasing intracellular calcium fluxes relying on a SMAD4-dependent manner, and any disruption of this mechanism may underlie alterations in phenotype and function. The following analysis of de-regulated exosomal miRNAs and transfection experiments reveals miR-494-3p and miR-1260a as potential mediators of SMAD family member 4 (SMAD4)-associated de-regulated calcium fluxes. Taken together, PDAC-exosomes from cells with, but mainly from those without SMAD4 expression, create an immunosuppressive myeloid cell background by increasing calcium fluxes through the transfer of SMAD4-related differentially expressed miR-1260a and miR-494-3p [120].

### miR-155

CD14^+^HLA-DR^low^ MDSCs accumulate in patients with B cell-derived chronic lymphocytic leukemia (CLL) and induce immune defects that prevent an efficient anti-tumor response. A previous study has indicated that MDSCs inhibit T cell responses in an indoleamine-2,3-dioxygenase (IDO)-dependent manner in CLL, and CLL-cells accelerate both the accumulation and activation of MDSCs. However, the underlying mechanism leading to a CLL-triggered reprogramming of regular monocytes to MDSCs remains unclear. The work by Bruns H et al. demonstrated that exosomes from CLL cells could be taken up by monocytes and then induce IDO-expressing MDSCs via the STAT1 pathway. In this process, a high level of exosomal miRNA-155 was transferred into monocytes and resulted in marked downregulation of 39 target genes of miR-155. Additionally, the exosomal miR-155 was found to induce MDSCs and enhance their suppressive function, suggesting that an exosomal transfer of miR-155 contributes to CLL-mediated MDSC induction [121].

### miR-126-3p, miR-27b, miR-320, and miR-342-3p

Ridder K et al. recently established a Cyclization Recombination Enzyme (Cre)/locus of X-overP1 (LoxP) system to trace exosomal RNAs from hematopoietic cells to neurons under inflammatory conditions. Tumor cells were stably transduced to constitutively express Cre recombinase and green fluorescent protein (GFP). After transplantation into a Cre reporter mouse, lateral transfer of Cre mRNA containing exosomes led to recombination in the host. It was indicated that > 90% of all recombined cells, in or around the tumor mass, were CD45^+^ leukocytes and about 50% were CD11b^+^Gr1^+^ MDSCs, demonstrating that MDSCs were principle recipient cells of TEXs. They also found that MDSCs internalizing labeled-TEXs displayed enhanced expression of suppressive molecules and altered miRNA expressing profile, including aberrant expression of miR-126-3p, miR-27b, miR-320, and miR-342-3p, which have been reported in the context of tumor progression [122].

### miR-29a and miR-92a

Results from the study by Guo X et al. demonstrated that TEXs from glioma could also enhance the expansion and suppressive function of MDSCs, both in vitro and in vivo, and hypoxia-induced TEXs exhibited a stronger ability for inducing MDSCs than did normoxia-induced TEXs. A following miRNA sequencing analysis of hypoxia-induced TEXs revealed that hypoxia-induced exosomal miR-29a and miR-92a expression resulted in the expansion of MDSCs. miR-29a and miR-92a conveyed by TEXs activated the proliferation and function of MDSCs by targeting HMGB1 and protein kinase cAMP-dependent type I regulatory subunit alpha (Prkar1a), respectively. In addition, the expression of miR-92a in TEXs accelerated the immunosuppressive function of MDSCs, while miR-29a only partially contributed to the suppressive function. Altogether, the study suggests that TEXs from glioma mediate the expansion and function of myeloid-derived suppressor cells through microRNA-29a/HMGB1 and microRNA-92a/Prkar1a pathways [123].

## Clinical application of TEXs

The most potent application of TEXs in clinic is their use as diagnostic and prognostic bio-markers. Released TEXs can be found in various body fluids and likely reflect the status of the parental cancer cells, implying that TEXs are ideal non-invasive bio-markers for cancer diagnosis. For instance, TEXs expressing CD63 and caveolin-1 in plasma can perform as non-invasive markers of melanoma, and reflect the clinical management of cancer patients [129]. Previously, no specific markers for distinguishing TEXs from normal exosomes have been known. However, a recent study identified exosomes derived from pancreatic cancer cells were enriched with a cell surface proteoglycan, glypican-1 (GPC1). Detection of GPC1^+^ circulating TEXs in the serum of patients with pancreatic cancer distinguished healthy donors and patients with a benign pancreatic disease from patients with early- and late-stage pancreatic cancer with high sensitivity and specificity. Moreover, amounts of GPC1^+^ circulating TEXs were positively correlated with tumor burden and the survival of pre- and post-surgical patients. GPC1^+^ circulating TEXs from patients with spontaneous pancreatic tumors carried specific KRAS mutation, and reliably reflected pancreatic intraepithelial lesions in spite of negative signals by magnetic resonance imaging. Therefore, GPC1^+^ circulating TEXs could be used as a highly specific bio-marker for pancreatic cancer, to detect early stages of pancreatic cancer and facilitate possible curative surgical therapy [130]. Besides that, a miRNA signature in circulating TEXs was found to be superior to exosomal GPC1 or plasma CA-199 level in diagnosing pancreatic cancer and identifying PDAC and pancreatic disease from chronic pancreatitis (CP) [131].

Exosomal ncRNAs have also been characterized as potential diagnostic and prognostic bio-markers [132]. In a research by Eichelser C et al., it was demonstrated that the level of exosomal miR-373 was specifically increased in the serum of triple-negative breast cancer patients and was linked to more aggressive tumors [133]. Additionally, exosomal miR-1290 and miR-375 upregulation may indicate poor overall survival in castration-resistant prostate cancer, and exosomal miR-19a level in serum is correlated with recurrence in colorectal cancer [134]. In another study, a xenograft model of acute myelocytic leukemia (AML) was developed and levels of a series of miRNAs in circulating exosomes, including let-7a, miR-99b, miR-146a, miR-155, miR-191, and miR-1246, showed a significant difference in leukemia-engrafted mice. Furthermore, it was revealed that levels of these miRNAs in circulating exosomes were markedly higher in AML patients compared to that in healthy individuals [135]. In prostate cancer (PC), exosomal miR-141 is found to be remarkably stable in the serum, which can better distinguish metastatic PC patients from healthy individuals with significant specificity and sensitivity [136].

Beside exosomal miRNAs, exosomal lncRNAs also act as potential biomarkers in cancer diagnosis. In a recent study, high lncRNA CRNDE-p and low miR-217 in TEXs were found to be correlated with tumor classification (T3/T4), clinical stage (III/IV), and lymph node or distant metastasis [137]. Similarly, exosomal lncRNAs, including Hox transcript antisense intergenic RNA (HOTAIR), metastasis-associated lung adenocarcinoma transcript 1 (MALAT1), and maternally expressed gene 3 (MEG3), are predominantly observed in cervical cancer–derived exosomes in cervicovaginal lavage samples. Levels of these lncRNAs are different in the cervicovaginal lavage samples of cervical cancer patients and cancer-free volunteers, indicating the potential for these exosomal lncRNAs to serve as bio-markers in the early diagnosis of cervical cancer [138]. Moreover, the combined detection of exosomal miR-21 and lncRNA HOTAIR can reflect the clinical stages of laryngeal squamous cell carcinoma and also perform as diagnostic bio-markers with high sensitivity and specificity [139]. Exosomal lncRNA zinc finger antisense 1 (ZFAS1) was evaluated in serum exosomes of gastric cancer patients. The increased exosomal lncRNA ZFAS1 level was significantly correlated with lymphatic metastasis and TNM stages [140]

Recently, circRNAs, which have a covalent loop structure that confers resistance to RNA exoribonuclease and own potential to regulate gene expression associated with tumor progression, have been identified to be enriched in TEXs compared to the producer cells [141]. Furthermore, the loading of circRNAs into exosomes may be regulated by associated miRNAs in producer cells. To date, more than 1000 circRNAs have been identified in human serum exosomes. CircRNAs originating from human cancer xenografts can enter the circulation and be readily measured in the serum. Intriguingly, serum exosomal circRNAs, such as circ-KLDHC10, are able to distinguish patients with colon cancer from healthy controls, indicating that exosomal circRNAs are potential diagnostic bio-markers for cancer [37]. All the clinical applications of TEX-molecules as bio-markers in informing the presence of malignant disease and tumor burden discussed above are implied in Table 3.

1. However, there still exist some key issues needed to be solved for further understanding of the role of TEXs in cancer diagnosis. The biggest challenge for TEX application in liquid biopsies is their isolation. Current exosome isolation methods include size-based isolation, ultracentrifugation, immune-affinity capture, water excluding polymer-based methods, and microfluidic-based platforms, solely or in combination. These techniques take advantage of structural features associated with exosomes, containing density, size, shape, and surface markers [142, 143]. However, ultracentrifugation, which is based on size isolation, still accounts as the “gold standard” technique for exosome isolation [128, 144]. Nevertheless, these methods still cannot confirm the purity and homogeneity of isolated TEXs, since different cancer cells deliver distinct exosomes. In addition, studies about MDSC-exosomes imply that exosomes from G-MDSCs can promote colorectal cancer cell stemness via exosomal S100A9 [145]. This finding revealed a direct regulation of MDSCs to cancer cells. However, there still lack specific markers to identify exosomes from MDSCs and different cancer cells.

**Table 3:** The application of TEXs in clinical diagnosis

TEX-molecule	Type of cancer	TEXs source	Application	Refs
CD63	Melanoma	Plasma	Diagnosis and prognosis	[129]
Caveolin-1				
GPC1	Pancreatic cancer	Serum	Early diagnosis	[130]
miR-373	Triple-negative breast cancer	Serum	Diagnosis and prognosis	[133]
miR-1290	Prostate cancer	Plasma	Therapy monitoring	[134]
miR-375				
miR-19a	Colorectal cancer	Serum	Prognosis	[134]
let-7a	AML	Serum	Early diagnosis	[135]
miR-99b				
miR-146a				
miR-155				
miR-191				
miR-1246				
miR-141	Prostate cancer	Serum	Early diagnosis	[136]
lncRNA CRNDE-p	Colorectal cancer	Serum	Diagnosis and prognosis	[137]
miR-217				
lncRNA HOTAIR	Cervical cancer	Cervicovaginal Lavage Samples	Early diagnosis	[138]
lncRNA MALAT1				
lncRNA MEG3				
miR-21	Lung cancer	Serum	Early diagnosis	[139]
lncRNA HOTAIR				
lncRNA ZFAS1	Gastric cancer	Serum	Early diagnosis	[140]
circ-KLDHC10	Colorectal cancer	Serum	Early diagnosis	[37]

Implies the clinical application of TEX-molecules as biomarkers in informing the presence of malignant disease and tumor burden

## Conclusions

In this review, we focused on the role of TEXs in regulating cell biology of MDSCs by conveying functional components. As described above, the package of functional components into TEXs is not random. In this process, TME promotes the formation and release of TEXs. Released TEXs then facilitate the expansion and function of MDSCs through transporting different cargoes. Expanded MDSCs promote the tumor progression through producing suppressive molecules. All these identify that exosomes are crucial for intercellular communication between cancer cells and MDSCs in TME.

## Data Availability

The material supporting the conclusion of this review has been included within the article.

## Abbreviations

Ago2: Argonaute 2
ALIX: ALG-2-interacting protein X
AML: acute myelocytic leukemia
Arg1: arginase 1
Bcl-xL: B cell lymphoma-extra large
BM: bone marrow
BMSCs: bone marrow stromal cells
C/EBP-β: CCAAT/enhancer-binding protein-β
CAFs: carcinoma associated fibroblasts
circRNAs: circular RNAs
CLL: chronic lymphocytic leukemia
Cox2: cyclo-oxygen-ase 2
CP: chronic pancreatitis
Cre: Cyclization Recombination Enzyme
CREB: cAMP-response element-binding protein
DAMPs: damage-associated molecular patterns
DCs: dendritic cells
Dsg2: desmoglein 2
ECM: extracellular matrix
EEs: early endosomes
EGFR: epidermal growth factor receptor
EGFRvIII: epidermal growth factor receptor vIII
EMT: epithelial-mesenchymal transition
ER: endoplasmic reticulum
Erk: extracellular regulated protein kinase
ESCRT: endosomal-sorting complex required for transport
EVs: extracellular vesicles
FTSECs: fallopian tube secretory epithelial cells
FYVE: Fab1p-YOTB-Vps27p-EEA1
GAS5: growth arrest specific 5
GBM: glioblastoma multiforme
G-CSF: granulocyte colony-stimulating factor
GDP: guanosine diphosphate
GFP: green fluorescent protein
GM3: monosialodihexosylganglioside
GM-CSF: granulocyte-macrophage colony-stimulating factor
GPC1: glypican-1
GTPases: guanosine triphosphate
H-GDEs: hypoxia-induced glioma exosomes
HGS: hepatocyte growth factor–regulated tyrosine kinase substrate
HIF-1α: hypoxia-inducible factor-1α
HLA-DR: human leukocyte antigen DR
HMGB1: high mobility group box 1
hnRNP: heterogeneous nuclear ribonucleoprotein
HOTAIR: Hox transcript antisense intergenic RNA
HPCs: hematopoietic progenitor cells
Hrs: hepatocyte growth factor–regulated tyrosine kinase substrate
Hsp70: heat shock proteins 70
ICD: immunogenic cell death
IDO: indoleamine-2,3-dioxygenase
IFN-γ: interferon-γ
IL: interleukin
ILVs: intraluminal vesicles
IMCs: immature myeloid cells
iNOS: inducible nitric oxide synthase
IRF8: interferon regulatory factor
JAK: janus kinase
LDH: lactate dehydrogenase
lncRNAs: long non-coding RNAs
LOX-1: lectin-type oxidized LDL receptor 1
LoxP: locus of X-overP1
MALAT1: metastasis associated lung adenocarcinoma transcript 1
MAPK: mitogen-activated protein kinase
Mcl-1: myeloid cell leukemia sequence 1
M-CSF: macrophage colony-stimulating factor
MDSCs: myeloid-derived suppressor cells
MEG3: maternally expressed gene 3
MHC: major histocompatibility complex
miRISC: RNA-induced silencing complex
miRNA: microRNA
MM: multiple myeloma
M-MDSCs: monocytic myeloid-derived suppressor cells
mRNAs: messenger RNAs
MSC: mesenchymal stem cells
mTOR: mammalian target of rapamycin
MVBs: multivesicular bodies
MVEs: multivesicular endosomes
MyD88: myeloid differentiation factor 88
Mϕ: macrophage
ncRNAs: non-coding RNAs
NF-κB: nuclear factor-kappa B
NK: natural killer cell
NOS2: nitric oxide synthase 2
NOX2: NADPH oxidase 2
nSMase2: neutral sphingomyelinase 2
OSCC: oral squamous cell carcinoma
PC: prostate cancer
PDAC: pancreatic cancer
PGE2: prostaglandin E2
PHD1/2: prolyl hydroxylase 1/2
PKM2: pyruvate kinase type M2
PMN-MDSCs: polymorphonucler myeloid-derived suppressor cells
Prkar1a: protein kinase cAMP-dependent type I regulatory subunit alpha
PSA: prostate specific antigen
PSAP: presenilin-associated protein
PtdIns3P: phosphatidyl inositol 3-phosphate
PTEN: phosphatase and tensin homolog
RORα: RAR-related orphan receptor alpha
ROS: reactive oxygen species
SCC: squamous cell carcinoma
SCF: stem cell factor
SMAD4: SMAD family member 4
SNAP-23: synaptosome-associated protein 23
SNARE: soluble N-ethylmaleimide-sensitive fusion factor attachment protein receptor
STAT: signal transducers and activators of transcription
TAMs: tumor-associated macrophages
TEXs: tumor-derived exosomes
TFEB: transcription factor EB
TGF-β: transforming growth factor-β
TGN: trans-Golgi network
Th17: T helper cell 17
TLR2: Toll-like receptor 2
TME: tumor microenvironment
TNF: tumor necrosis factor
Tregs: regulatory T cells
TRIP: TRAF-interacting protein
TSAP6: the transmembrane protein tumor suppressor-activated pathway 6
TSG101: tumor susceptibility gene 101
UBR2: ubiquitin protein ligase E3 component n-recognin 2
UCA1: urothelial carcinoma-associated 1
UTR: untranslated region
VAMP: vesicle-associated membrane protein
VEGF: vascular endothelial growth factor
Vps4: vacuolar protein sorting protein 4
ZFAS1: zinc finger antisense 1
ZO-1: zona occludens protein 1
